# Endovascular treatment of small-parent artery aneurysms: mid-term results of the silk vista baby flow diverter

**DOI:** 10.1007/s00234-025-03653-7

**Published:** 2025-05-22

**Authors:** Celal Cinar, Alperen Elek, Mahmut Kusbeci, Egemen Ozturk, Cemre Yanbol Utli, Ismail Oran

**Affiliations:** Department of Interventional Radiology, Faculty of Medicine, Ege University, Izmir, Turkey

**Keywords:** Silk Vista Baby (SVB), Low profile flow diverter, Intracranial aneurysm, Small parent artery, Flow diverters

## Abstract

**Purpose:**

We aim to retrospectively evaluate patients treated with SVB at our single center, assessing its efficacy, radiologic and clinical outcomes, and complication profile.

**Method:**

This retrospective, single-center study included patients with small parent artery aneurysms treated with SVB. Patients were monitored for procedural success, aneurysm occlusion, and complications, with radiologic and clinical follow-up conducted.

**Results:**

The study included 64 patients with 66 small parent artery aneurysms treated using SVB FDs. Among these patients, 26 (40.6%) presented with subarachnoid hemorrhage (SAH). The mean index aneurysm size was 5.7 ± 5.25 mm. The cohort consisted of 15 males (23.4%) and 49 females (76.6%), with a mean age of 54.23 ± 14.95 years (range: 5–80 years). SVB was used as a standalone treatment in 42 patients (65.6%), while adjunctive materials were applied in 22 cases (34.4%)—coils in 16 (24.2%) and a regular stent in 6 (9.1%). A total of 71 SVB stents were deployed for 66 aneurysms, achieving a 100% technical success rate. In 30 patients with multiple aneurysms, additional aneurysms were addressed during the same session along with the index aneurysm. The mean duration of clinical and radiological follow-up was 10.5 ± 7.94 months. Among a total of 7 patients (10.9%) with ischemic complications, 3 (4.7%) were related to SVB implantation. Index aneurysm occlusion was accomplished in 93.3% of cases at the last follow-up. Favorable neurological outcomes (mRS 0–2) were recorded in 53 patients (82.8%). Subgroup analysis showed that 6 out of 7 total complications (85.7%) occurred in patients with multiple aneurysms.

**Conclusion:**

The SVB FDs are a highly effective and safe treatment option for distal intracranial aneurysms, achieving high occlusion rates with a favorable safety profile.

## Introduction

The endovascular treatment of intracranial aneurysms has undergone significant evolution in recent years. Flow diverters (FDs) have emerged as an effective therapeutic option, particularly for wide-neck and complex aneurysms [4, 7–9]. Traditional FDs were primarily designed for large-caliber vessels, while treating distal cerebral vessels remained challenging due to technical limitations. To address this, low-profile FDs have been developed to facilitate the treatment of small-parent artery aneurysms.

The Silk Vista Baby (SVB) is a low-profile FD stent designed for vessels ranging from 1.5 mm to 3.5 mm in diameter, deliverable through a 0.017” microcatheter. It offers high radiopacity, flexibility, and optimal pore density, making it a promising option for the treatment of distal cerebral aneurysms [1, 20]. The key advantages of SVB include its suitability for small-caliber vessels, high adaptability, and precise control during implantation. However, the long-term efficacy and safety of flow diverters in distal cerebral aneurysms remain areas of ongoing investigation [1, 2, 20–22]. Previous studies have demonstrated high technical success rates and acceptable complication rates with SVB use. However, these studies have been limited by small sample sizes, necessitating further research with radiological and neurologic outcome data to strengthen the existing literature.

In this study, we aim to retrospectively evaluate patients treated with SVB at our single center, assessing its efficacy, radiologic and clinical outcomes, and complication profile.

## Methods

### Study design and patient selection

This retrospective, single-center study includes cases of small parent artery (≤ 3.5 mm) aneurysms treated with the SVB FDs at our institution during the last 3 years. The study was approved by our Ethics Committee, and written informed consent was obtained from all patients.

Inclusion criteria encompassed patients with saccular, fusiform, blister, or dissecting aneurysms treated with SVB, confirmed by digital subtraction angiography (DSA) or magnetic resonance angiography (MRA). Patients with incomplete clinical and radiological data were excluded from the study. No age restrictions were applied during patient selection. Patients who had undergone prior treatment for other aneurysms were not excluded.

## Procedural technique

All unruptured patients received a single dose of a histamine H2-receptor antagonist along with a loading dose of 30–60 mg prasugrel and 300 mg aspirin 9–12 h before the procedure. In patients aged 65 years and older, ticagrelor 180 mg was used instead of prasugrel for loading. The efficacy of prasugrel and aspirin was confirmed just before the procedure using multiple electrode aggregometry (Multiplate; Roche Diagnostics, Munich, Germany). According to the opinion of the Working Group on High On-treatment Platelet Reactivity, an adenosine diphosphate (ADP) aggregation value > 47 U was considered non-responsiveness or low response (resistance) to antiplatelet therapy (the normal range reported by the manufacturer, in the absence of antiplatelet therapy, is 57–113 U) [23]. For patients with SAH, the loading dose consisted of 300 mg aspirin and 180 mg ticagrelor, administered via orogastric tubing under the protection of bridging intravenous tirofiban (a short half-life platelet glycoprotein IIb–IIIa receptor inhibitors) in a dose of 0.5–1 mg [17].

The procedures were performed under general anesthesia, with intravenous propofol used for induction and sevoflurane gas for maintenance. Systemic heparinization was initiated with an initial bolus of 5000–10,000 IU heparin, maintaining the activated clotting time (ACT) at two to three times the baseline level [6]. Post-procedurally, dual antiplatelet therapy was continued for at least 6 months, followed by aspirin monotherapy.

All interventions were performed via femoral artery access using a triaxial system. The SVB stent was selected based on the appropriate diameter and length and was delivered using a 0.017” microcatheter (Headway 17, Microvention Terumo, USA) and 0.008 inch guide wire (Asahi Chikai, Japan). Special attention was given to the precise positioning of the stent’s proximal and distal landing zones, particularly in tortuous or bifurcated segments, to minimize the risk of retrograde displacement or malposition, which was dynamically assessed via angiography. During the use of SVB, adjunctive materials and techniques were employed when deemed necessary by the operators based on their experience.

For cases with multiple aneurysms, priority was given to the index aneurysm. In patients with SAH, the ruptured aneurysm was treated first. If no complications arose, additional aneurysms were treated sequentially at the operator’s discretion. In these cases, the contemporary materials and techniques were utilized, including regular flow diverters, conventional stents, coils, or their combinations to achieve optimal treatment outcomes. Patients were monitored in the intensive care unit for 24 h to observe for early complications, including distal embolization and intracranial hemorrhage.

## Follow-up protocol and evaluation

Patients were closely monitored for early post-procedural neurological complications and underwent scheduled clinical and radiological follow-ups according to predefined protocols. Follow-up imaging, including CTA, MRA or DSA, was performed at 3-, 6-, and 12-months post-procedure. Aneurysm occlusion was graded using the O’Kelly-Marotta (OKM) scale. Symptomatic thromboembolic events, in-stent thrombosis, distal embolization, and intracranial hemorrhage were documented. Patients who experienced fatal outcomes were included in the calculation of periprocedural complication rates. However, for the assessment of aneurysm occlusion rates, three deceased patients and one patient with severe morbidity were excluded from the analysis.

Technical success was defined as the successful deployment of the stent in the planned position without requiring additional corrective interventions. Mid-term clinical outcomes were assessed using the modified Rankin Scale (mRS), with scores of 0–2 considered indicative of a favorable clinical outcome. Follow-up imaging was also evaluated for in-stent stenosis, with additional antiplatelet therapy or revascularization strategies implemented as necessary.

### Statistical analysis

Data analysis was conducted using R (version 4.1.2; The R Foundation for Statistical Computing, Vienna, Austria). Continuous variables were reported as mean ± standard deviation (SD), while categorical variables were summarized as frequencies (%). Group comparisons were performed using the independent t-test or Mann-Whitney U test for unpaired continuous variables and the paired t-test or Wilcoxon signed-rank test for paired continuous variables, as appropriate. Categorical variables were compared using the chi-square test or Fisher’s exact test. Logistic regression analysis was employed to identify factors associated with complications. A p-value of < 0.05 was considered statistically significant.

## Results

**Fig. 1:**
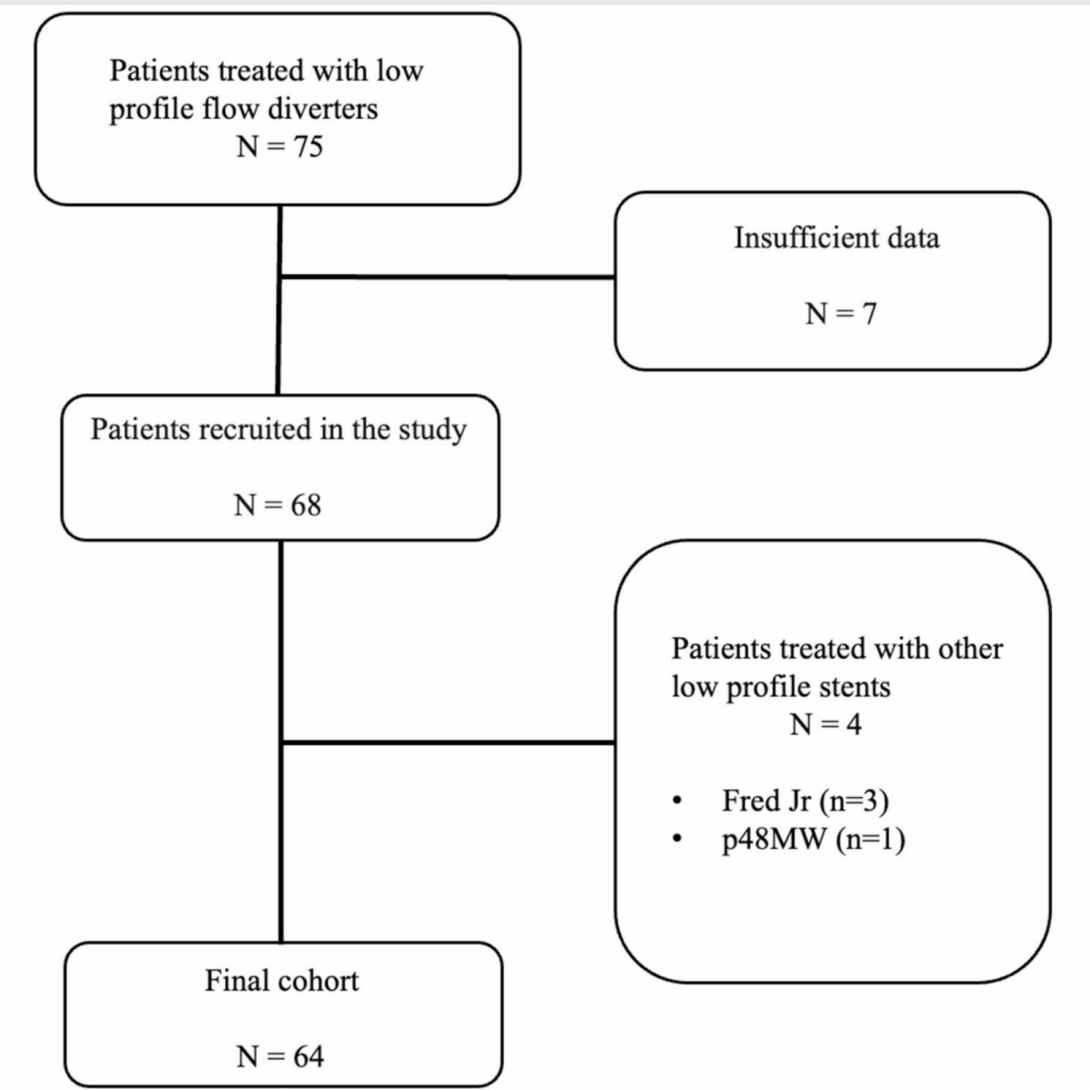
Flow diagram

**Fig. 2:**
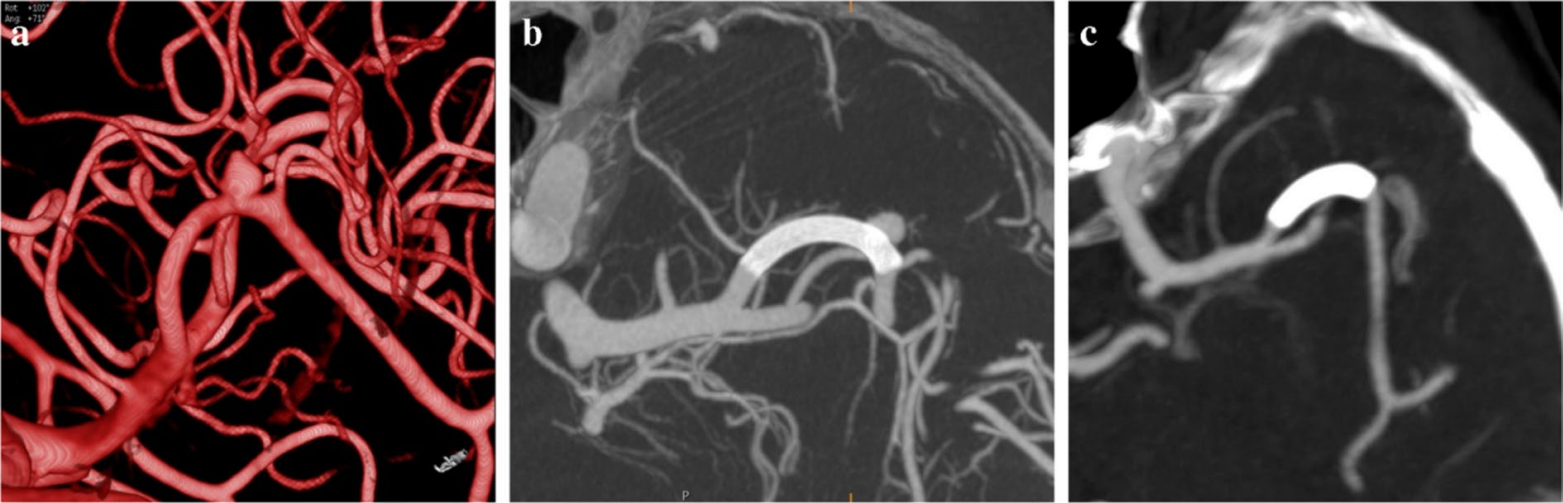
A 69-year-old female patient with a positive family history of cerebral aneurysm. (**a**) Axial 3D angiogram showing a saccular aneurysm located in the M2–3 segment of the left MCA. (**b**) Cone-beam CT obtained after SVB stent implantation. (**c**) Six-month follow-up CTA reveals disappearance of the aneurysm

**Fig. 3:**
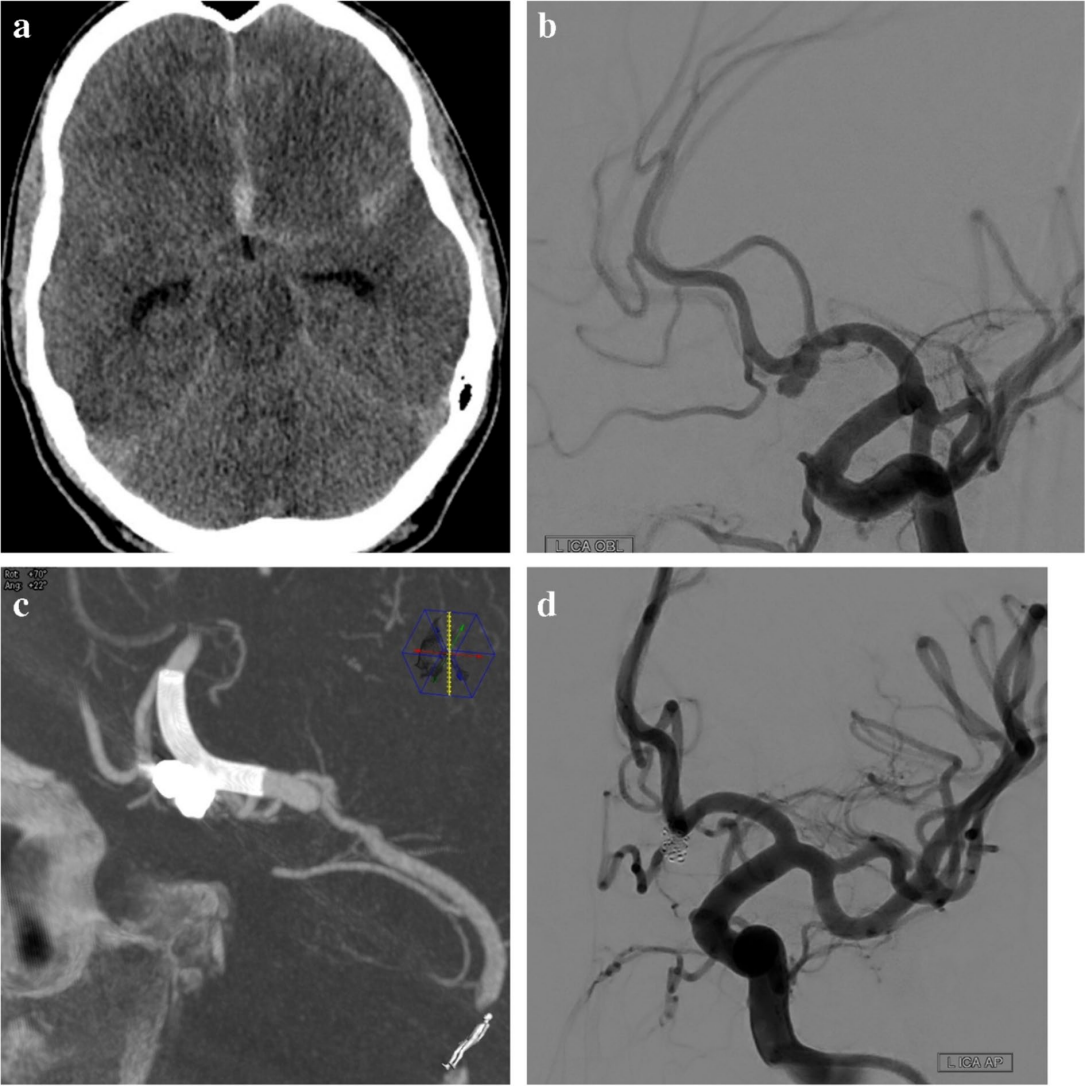
A 24-year-old male patient with subarachnoid hemorrhage (SAH). (**a**) CT demonstrating SAH predominantly in the anterior interhemispheric sulcus. (**b**) Left carotid angiogram in a left oblique projection revealing a multilobulated saccular aneurysm at the anterior communicating artery. (**c**) Cone-beam CT after embolization showing the multilobulated aneurysm filled with coils and the SVB stent positioned in the A1 and A2 segments of the ipsilateral ACA. (**d**) Twelve-month follow-up angiogram confirms occlusion of the aneurysm

**Fig. 4:**
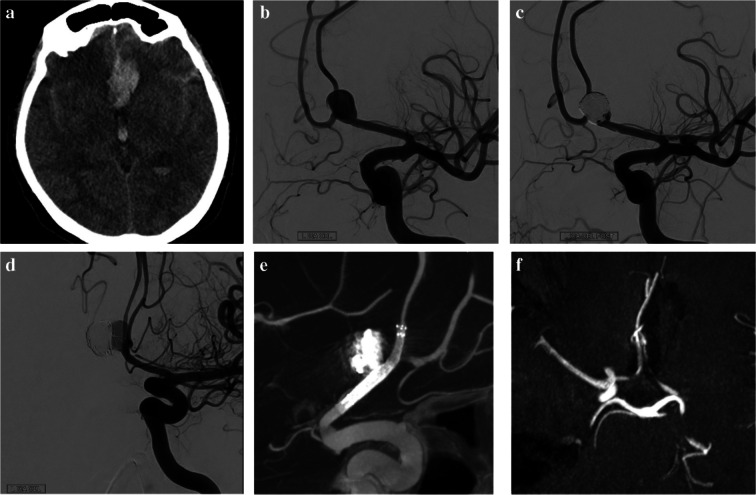
A 57-year-old female patient with SAH. (**a**) CT demonstrating cisternal and ventricular bleeding, along with a hematoma in the anterior interhemispheric sulcus. (**b**) Left carotid angiogram in a left oblique projection revealing a saccular aneurysm arising from the distal A1 segment of the left ACA. (**c**) After intrasaccular coil deposition with the assistance of an LVIS Evo stent deployed in the A1 and A2 segments of the left ACA, the final angiogram shows some residual filling. (**d**) Two and a half months later—15 days prior to the scheduled follow-up imaging—the patient experienced another SAH. A left carotid angiogram in a slight contralateral oblique projection reveals regrowth of the aneurysm. (**e**) Cone-beam CT imaging following placement of an additional LVIS Evo stent and subsequent SVB implantation. (**f**) MR examination six months later reveals no aneurysmal filling and patent parent vessels

**Fig. 5:**
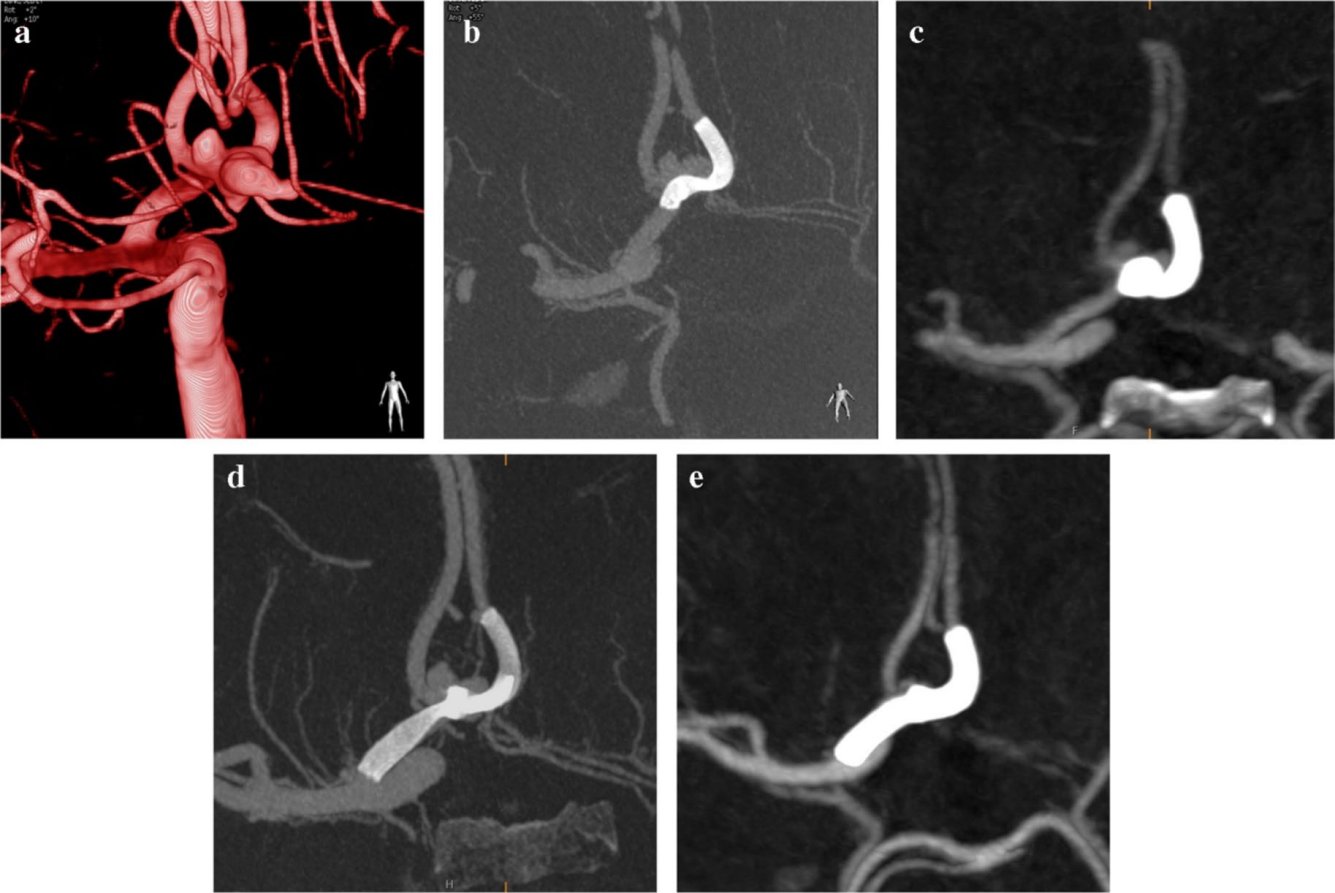
A 49-year-old male patient presented with an incidental saccular aneurysm. (**a**). A frontal 3D angiogram demonstrated a multilobulated saccular aneurysm arising from the ACom artery. (**b**) A cone-beam CT scan obtained after SVB stent implantation. (**c**) A six-month follow-up CTA revealed proximal shortening and deformation of the stent, along with contrast filling of the aneurysm. (**d**) A second SVB stent was subsequently implanted, and a cone-beam CT scan obtained after the procedure clearly demonstrated proximal deformation of the first stent and suboptimal expansion of the second stent. Fifteen months after the second treatment, follow-up CTA revealed some residual filling of the aneurysm, although marked shrinkage was evident

The study cohort included 64 patients with 66 small parent artery aneurysms treated with SVB FDs (Figs. 1, 2, 3, 4 and 5). The mean aneurysm size was 5.7 ± 5.25 mm. The cohort consisted of 15 male (23.4%) and 49 female (76.6%) patients, with a mean age of 54.23 ± 14.95 years (range: 5–80 years). Among these patients, 26 (40.6%) presented with SAH, including traumatic in one patient, rebleeding in one, and surgery-related iatrogenic in one. Seven patients with recurrent/residual aneurysm had previously undergone treatment for small parent artery aneurysms using various methods, including clipping (*n* = 1), Woven EndoBridge (WEB) device (*n* = 1), stent-assisted coiling (*n* = 1), regular FD (*n* = 1), and simple coiling (*n* = 3). In the remaining cases, the small parent artery aneurysm had not been previously treated. Key demographic and aneurysm characteristics are summarized in Table 1.

**Table 1:** Patient demographics and aneurysm characteristics

Variable	*n* (%) or Mean ± SD
*Patient Demographics*	
Total Patients	64
Age (years)	54.23 ± 14.95
Gender (Male/Female)	15/49
SAH	26/64 (40.6%)
Previous-treatment History	7 (10.9%)
Patients with Multiple Aneurysms	30/64 (46.8%)
*Aneurysm Characteristics*	
Total Aneurysms	122
Aneurysms Treated with SVB (index aneurysm)	66
*Index Aneurysm Morphology*	
Saccular	62/66 (93.94%)
Fusiform	4/66 (6.06%)
*Index Aneurysm Location*	
Acom	36 (54.54%)
DACA	14 (21.21%)
M2-3	4 (6.06%)
A1	4 (6.06%)
MCABif	2 (3.03%)
Basilar Fenestration	2 (3.03%)
Acho	1 (1.15%)
P1-2	1 (1.15%)
M3-4	1 (1.15%)
P2-P3	1 (1.15%)
*Pre-treatment Mean Aneurysm Size (mm)*	5.7 ± 5.25 mm

Acom: Anterior Communicating Artery, DACA: Distal Anterior Cerebral Artery, M2-3: Middle Cerebral Artery (MCA) segments M2 and M3, A1: A1 segment of the Anterior Cerebral Artery (ACA), MCABif: Middle Cerebral Artery Bifurcation, Acho: Anterior Choroidal Artery, P1-2: Proximal and mid-segment of the Posterior Cerebral Artery (PCA), M3-4: Distal segments of the Middle Cerebral Artery (MCA), P2-3: Distal segments of the Posterior Cerebral Artery (PCA), SAH: Subarachnoid Hemorrhage, SVB: Silk Vista Baby

SVB was used as a standalone treatment in 42 patients (63.6%), while adjunctive materials were utilized in 22 cases (34.4%), including coils in 16 cases (24.2%) and a regular stent in 6 cases (9.1%). A total of 71 SVB stents were deployed for the treatment of 66 aneurysms, achieving a 100% technical success rate. Branch artery jailing occurred in 17 patients (26.5%) following SVB stent placement (Table 2). Patients with multiple aneurysms underwent treatment for their index aneurysms and additional aneurysms in the same session. For these additional aneurysms, the treatment strategies included regular FD alone (*n* = 40), regular FD with coiling (*n* = 5), Y-stenting with coiling (*n* = 5), and stent-assisted coiling (*n* = 6).

**Table 2:** Treatment characteristics, clinical outcomes, and complications

Variable	*n* (%)
*Treatment Characteristics*	
Just SVB	42/64 (65.62%)
Complementary coiling	16/64 (25%)
Additional (Regular) stent	6/64 (9.37%)
Number of SVB used	71
Jailed branch (Yes/No)	17/47 (26.56%)
*Outcomes*	
Complete Index Aneurysm Occlusion	56/60 (93.3%)
mRS at last follow-up	mRS 0–2 (*n* = 53, %82.81)
	mRS 3–4 (*n* = 5, %7.81)
	mRS 5 (*n* = 3, %4.68)
	mRS 6 (*n* = 3, %4.68)
All-cause- Complications	7/64 (10.93%)
Just SVB related Complications	3/64 (4.68%)
All-cause-Mortality	3/64 (4.68%)
Re-treatment necessities	1/60 (1.66%)

The mean duration of clinical and radiological follow-up was 10.5 ± 7.94 months. Ischemic complications occurred in 7 patients (10.9%); 3 of 7 (4.7%) were related to SVB implantation. These included jailed artery occlusion, anterior cerebral artery (ACA) thrombosis, and posterior cerebral artery (PCA) dissection, the latter of which was successfully treated with a regular stent.

Complete index aneurysm occlusion was achieved in 93.3% of cases. Whereas 4 patients experienced partial occlusion, all of whom were treated solely with SVB without adjunctive materials. Favorable neurological outcomes (mRS 0–2) were recorded in 82.8% of patients at discharge. Three patients had a fatal outcome, and one patient required retreatment (Table 2).

The retreated patient initially underwent SVB placement, but the aneurysm remained patent at the 6-month follow-up. Upon reevaluation, it was discovered that the proximal portion of the stent had shortened, preventing aneurysm occlusion. A new SVB was placed, but even after 15 months, the aneurysm remained unoccluded (Fig. 5).

Among the three fatal SAH cases, two patients experienced periprocedural in-stent thrombosis and subsequent vessel infarction—one involving the SVB in the ACA and the other involving a regular stent in the PCA. These ischemic complications, combined with SAH, were considered contributing factors to the patients’ deaths. In the third fatal case, the implanted SVB remained patent until the patient’s passing.

Subgroup analyses were conducted based on the presence of multiple aneurysms, presence of SAH, and use of adjunctive materials (Tables 3, 4 and 5). Notably, 6 out of 7 total complications (85.7%) occurred in patients with multiple aneurysms. Logistic regression analysis indicated that the presence of multiple aneurysms was a near-significant predictor of complication risk (*p* = 0.072). Aneurysms treated with adjunctive materials had a significantly larger diameter compared to those treated with SVB alone (*p* = 0.007).

**Table 3:** Subgroup analysis between the patient’s aneurysm status

	Patient with multiple aneurysm (*n* = 30)	Patient with Single Aneurysm (*n* = 34)	*P* value
Age	57.33 ± 12.6	51.5 ± 16.45	0.057
Aneurysm (treated with SVB) Size	4.65 ± 2.52	6.67 ± 6.79	0.056
Treatment Modalities	SVB (*n* = 24)	SVB (*n* = 22)	0.094
	SVB + adjunctive Materials (*n* = 6)	SVB + Adjunctive Materials (*n* = 16)	
All-cause complication	6/30 (20%)	1/34 (2.94%)	**0.044**
Favorable Neurologic Outcome	23/30 (76.6%)	30/34 (88.24%)	0.372
Complete Occlusion*	24/26 (92.3%)	32/34 (94.1%)	0.776

*Since occlusion follow-up data was not available for 4 patients, calculations were performed based on a total of 60 patients

**Table 4:** Subgroup analysis between the patient’s SAH status.

Variable	Subarachnoid Hemorrhage (*n* = 26)	No Hemorrhage (*n* = 38)	*P* value
Age	50.15 ± 18	57.02 ± 11.91	**0.047**
Index Aneurysm Size	2.8 ± 5.32	6.5 ± 5.97	0.58
Treatment Modalities	SVB (*n* = 10)	SVB (*n* = 32)	**< 0.001**
	SVB + adjunctive Material (*n* = 16)	SVB + adjunctive Materials (*n* = 6)	
Multiple Aneurysm	Yes (*n* = 12)	Yes (*n* = 18)	0.923
	No (*n* = 14)	No (*n* = 20)	
All-cause complication	3/26 (11.54%)	4/38 (10.53%)	0.898
Favorable Neurologic Outcome	17/26 (65.38%)	36/38 (94.73%)	**0.004**
Complete Occlusion*	22/22 (100%)	34/38 (89.4)	0.286

*Since occlusion follow-up data were not available for 4 patients, calculations were performed based on a total of 60 patients

**Table 5:** Subgroup analysis between the patient’s adjunctive material using status.

	Just SVB (*n* = 42)	SVB + Adjunctive Materials (*n* = 22)	*P* value
Age	55.88 ± 13.32	51.09 ± 17.55	0.134
Aneurysm Size	4.2 ± 2.34	8.47 ± 7.64	**0.007**
All-cause complication	4/42 (9.5%)	3/22 (13.6%)	0.671
Favorable Neurologic Outcome	35/42 (83.3%)	18/22 (81.8%)	0.878
Complete Occlusion*	37/41 (90.2)	19/19 (100%)	0.297

*Since occlusion follow-up data was not available for 4 patients, calculations were performed based on a total of 60 patients

The SAH group had a significantly higher rate of adjunctive material use compared to the unruptured aneurysm group (*p* < 0.001). Although there was no significant difference in complication rates between the two groups, neurological outcomes were significantly better in the unruptured aneurysm group (*p* = 0.004).

## Discussion

This study demonstrates that the use of SVB FDs for the treatment of small parent artery aneurysms is associated with high technical success and favorable mid-term clinical outcomes. SVB placement was technically successful in 100% of cases, with an overall aneurysm occlusion rate of 93.3% at last follow-up. The incidence of ischemic complications was 10.93%, while SVB-related complications were observed in only 4.69% of patients.

One of the most critical advantages of the SVB is its compatibility with a 0.017‑inch microcatheter, which facilitates access to challenging vascular territories such as distal cerebral arteries. The low crossing profile of SVB not only enables access to small and distal vessels but also contributes to procedural safety by minimizing vascular manipulation, facilitating smoother navigation, and reducing the risk of vasospasm. The device’s 48‑wire braided structure enhances flexibility while providing high radiopacity, thereby making the deployment process safer. Several studies have underscored these characteristics, reporting high technical success rates with minimal complications [15, 16, 18, 19]. Moreover, the SVB’s ability to navigate tortuous vascular anatomy renders it particularly suitable for cases in which larger-caliber conventional flow diverters are not viable.

In terms of efficacy, previous studies have reported occlusion rates ranging from 57.1 to 94% at follow-up periods of up to 12 months [10–12, 14]. Our findings align with this trend, as we observed a final occlusion rate of 93%. Notably, adjunctive materials were utilized in 22 cases (16 cases with coiling and 6 cases with an additional stent), which likely contributed to the high occlusion rates. It is important to note that all four cases of partial occlusion were treated solely with the SVB, without adjunctive materials. Although previous studies have reported the use of adjunctive materials in complex cases, they have not provided detailed subgroup analyses; our study helps to fill this gap.

The use of the SVB in ruptured aneurysms remains a topic of debate. While some studies suggest favorable outcomes, early rebleeding and thrombotic complications continue to be a concern. Although flow diverters inherently have a higher metal coverage compared to conventional stents and are therefore theoretically associated with an increased risk of thrombotic complications, the antiplatelet management strategies are similar in the acute rupture setting regardless of the stent type. Importantly, the use of newer-generation ADP receptor blockers such as prasugrel or ticagrelor, bridging therapy with intravenous antiplatelet agents like tirofiban during on-table loading when necessary, and peri-procedural monitoring with platelet aggregometry can effectively mitigate thrombotic risks. Adjusting therapy based on aggregometry findings during the early perioperative period allows close monitoring and timely intervention to prevent thrombotic events. These strategies have been shown to reduce thrombotic complication rates to acceptable levels even after flow diverter placement for ruptured blister aneurysms, as previously demonstrated in the literature [5].

Russo et al. [20] reported that the SVB shows promise for the treatment of ruptured intracranial aneurysms, although worse clinical outcomes were observed in posterior circulation cases. In their series of 25 patients with ruptured aneurysms, 79.1% achieved a favorable outcome (mRS 0–2), with a mortality rate of 12.5% and complete occlusion achieved in 85.7% at a 3‑month follow-up. In our study, 40.6% of cases presented with SAH, and subgroup analysis further revealed that although complication rates did not significantly differ between the SAH and No-SAH groups, neurological outcomes were worser in SAH patients. Similarly, in the study by Maybaum et al. [16] of 31 SAH patients, 18 achieved complete recovery (GOS 5), 4 had moderate disability (GOS 4), 2 had severe disability (GOS 3), 1 was in an unresponsive wakefulness syndrome (GOS 2), and 6 died (GOS 1).

Another key factor influencing outcomes is the presence of multiple aneurysms. Distal aneurysms are generally not found in isolation; they more frequently coexist with additional aneurysms, which often necessitate the use of additional stents or adjunctive materials. This situation makes it challenging to isolate the outcomes specifically attributable to the SVB. Our subgroup analyses addressed this issue by demonstrating that among patients with isolated distal aneurysms, the overall complication rate was significantly lower, with only one patient experiencing a complication. Although previous studies have not conducted detailed subgroup analyses to highlight this factor, our findings suggest that it can have a direct impact on outcomes.

In patients with multiple aneurysms, one-stage treatment was selectively employed based on individualized risk assessment, anatomical characteristics, and clinical factors. Literature supports that one-stage intervention can prevent rebleeding from untreated aneurysms, alleviate patient anxiety, and reduce the risks and costs associated with multiple procedures [3, 13, 24]. Although staged treatment may offer a lower risk of immediate procedural complications, in cases where the ruptured aneurysm could not be reliably identified, or multiple aneurysms were considered high-risk, one-stage treatment was regarded as both appropriate and safe. Our approach is consistent with these findings, further highlighting the importance of patient-specific treatment planning.

Another important consideration is that, in patients treated with FDs, there is a worldwide trend to use adjunctive materials to fill the aneurysm sac as the aneurysm diameter increases. This strategy is aimed at reducing recurrence by promoting complete occlusion of the aneurysm. In our patient population, we observed a similar pattern, with larger aneurysms more frequently receiving adjunctive coiling or additional stenting to achieve optimal long-term outcomes.

Although the radiological follow-up of jailed branch arteries was not a specific objective of this study, it is known that many crossed side branches become asymptomatically occluded over time, particularly when distal collateral flow is adequate. In our cohort, a side branch was crossed in 17 patients, yet only one of these cases (5.88%) resulted in a clinically evident thrombotic complication.

Although the SVB is a leading low-profile FD for treating distal aneurysms, our study did not include or compare other low-profile FDs such as Fred Jr. or p48 MW. A recent meta-analysis by Elek et al. [10] have compared low-profile FDs for distal aneurysms. Overall, the indicated that ruptured aneurysms had higher complication rates (20%) than unruptured ones (12.7%). In the case of SVB, the complication rate was 15%, with 17.4% in the SAH group and 12.2% in the incidental group. In our study, the overall complication rate was 10.93%, and 11.54% in the SAH group, both of which are slightly better than those reported in the meta-analysis.

Our study’s primary limitations include its retrospective design and relatively small sample size, which may affect the generalizability of our findings. Although our results are promising, particularly in complex aneurysm cases, longer-term follow-up is required to fully assess the durability of SVB treatment. Future prospective multicenter studies with standardized antiplatelet management and imaging follow-up protocols are essential to better define the role of SVB in the treatment of intracranial aneurysms.

## Conclusion

Our findings support the feasibility of SVB as an effective and safe treatment option for distal intracranial aneurysms, achieving high occlusion rates with a favorable safety profile. However, optimal outcomes require careful patient selection, precise technical execution, and long-term follow-up to ensure sustained efficacy and safety.

## Data Availability

No datasets were generated or analysed during the current study.
